# Are active video games useful in the development of gross motor skills among non-typically developing children? A meta-analysis

**DOI:** 10.1186/s13102-022-00532-z

**Published:** 2022-07-23

**Authors:** Sen Li, Yang Song, Zhidong Cai, Qingwen Zhang

**Affiliations:** 1 School of Physical Education, Shanghai University of Sport, Hengren Road 200, Shanghai, 200438 China; 2 School of Physical Education and Health, Shanghai Lixin University of Accounting and Finance, Shanghai, 201620 China; 3 Rizhao Sports School, Rizhao, 276800 China

**Keywords:** Active video games, Gross motor skill, Non-typically developing children, Meta-analysis

## Abstract

**Background:**

Proficiency in gross motor skills (GMS) lays the foundation for developing more complex motor skills. Improving these motor skills may provide enhanced opportunities for the development of a variety of perceptual, social, and cognitive skills. However, GMS development and intervention effects are not ideal for many non-typically developing children.

**Objective:**

To systematically evaluate the effect of active video games on the development of gross motor skills in non-typically developing children and adolescents.

**Methods:**

Seven Chinese and English databases were searched for randomized controlled trials, and the risk of bias in included studies were qualitative evaluation according to the revised Cochrane risk of bias tool for randomised trials (RoB 2). Then a meta-analysis was conducted to estimate the overall effect of active video games on the development of gross motor skills in non-typically developing children.

**Results:**

Twenty papers were included. In the three subordinate concepts of gross motor skills, active video games significantly improved locomotor skills [ SMD = 0.59, 95% CI (0.40, 0.77)] and non-locomotor skills [SMD = 0.51, 95% CI (0.20, 0.81)] in non-typically developing children. However, there was no significant difference compared with the control group [ SMD = 0.32, 95% CI (− 0.17, 0.82)] in object control skills.

**Conclusions:**

The study shows that active video games can improve locomotor skill and stability skill in non-typically developing children, but the effect on object control skill is uncertain, and more high-quality literature needs to be included in the future.

*Trial registration* The meta-analysis was registered on INPLASY (202,250,124) and is available in full on inplasy.com (https://inplasy.com/inplasy-2022-5-0124/).

## Background

Motor development is one of the most basic and important areas of individual development [1]. In general, there are relatively predictable sequential motor development stages and milestones in typically developing children [2, 3]. However, motor skill deficits caused by developmental disabilities, such as cerebral palsy (CP), developmental coordination disorder (DCD), and Down syndrome (DS), are common in non-typically developing children (NTDC). These children are often accompanied by various degrees of damage to the brain or central nervous system disorders, manifesting in delayed development and deficits in balance and movement skills, and gross and fine motor development fall behind typically developing peers [4].

Motor skill deficits, especially gross motor skill (GMS) disorder, are an important factor hindering children’s participation in physical activities; they increase sedentary time, affect their physical activity level and weight status [5], and pose great risks to their health. Individuals with motor skill deficits not only fall behind their peers in strength, coordination and endurance but also face the risk of many mental diseases, such as depression and social phobia [6]. Therefore, it is very important to pay attention to the development of GMSs in NTDC.

Motor skill intervention is often provided to develop the gross motor function of NTDC [7]. However, traditional motor intervention therapy often requires the help of various games and facilities and requires a large activity space and experienced therapists to accurately control the treatment process to ensure the participants' interest in the treatment process and the smooth progress of the treatment [8]. Most importantly, the level of motor skills among NTDC is poor, and it is difficult to maintain adherence to and motivation for the highly structured and repetitive activities of traditional rehabilitation [9]. A potential area of improvement for such interventions may lie in the attractiveness of play and children's preference for and participation in technology [10].

Active video games (AVGs; also known as exergames) have been proposed as a good alternative for traditional exercise and have become an emerging tool for developing motor skills using the new technologies [11] and developing such skills among NTDC [12–15]. AVG is a kind of sports entertainment games with the help of human–computer interaction, motion sensing, virtual reality and other high-tech technologies [16], require players to physically interact with on-screen avatars through various physical activities such as dancing, jogging, and boxing [17, 18]. AVGs can provide an ecological environment similar to that in the real world, where participants can practice specific tasks, and the difficulty of tasks can be adjusted readily in the game and provide sufficient challenges [4]. Such an immersive experience in a safe, enjoyable, and playful environment is associated with less fatigue and more relaxation, which may be attractive to children [19]. Simultaneously, the characteristics and animation effects of the game can also increase children’s motivation and participation in the gaming process, entice users to immerse themselves in the sports environment [20], and improve their cognitive function and motor skills. Hence, AVGs are suitable as rehabilitation tools for children and have gradually developed into a popular therapy of motor skill intervention for special populations [21].

Limited by game platforms, disease types, instruction degree, exercise dose, etc., there are differences in the intervention effect of AVGs on GMS of NTDC, and there is a lack of sufficient quantitative research to support the intervention effect. The current study focuses on how AVGs can improve motor skills among NTDC. If there are no certain answers to the above concern, the promotion and application of AVGs in the field of medical rehabilitation will be greatly restricted. GMS refers to the movement generated by large muscles or muscle groups of the body, including walking, running, jumping, throwing, etc. According to the change in spatial position and the control of external tools, GMS can be divided into locomotor skills (LS), object control skills (OCS) and non-locomotor skills (NLS) [22]. The purpose of this study was to explore the intervention effect of AVGs on the GMS of NTDC and to explore the dose effect from the aspects of the game platform, intervention setting, intervention duration, intervention frequency and intervention cycle to provide a reference for sports intervention and clinical research on the GMS of NTDC in the future.

## Methods

This study followed the requirements of the Preferred Reporting Items for Systematic Reviews and Meta-Analyses (PRISMA) 2020 statement [23] for the selection and use of research methods. The protocol for this systematic review was registered on INPLASY (202,250,124) and is available in full on inplasy.com (https://inplasy.com/inplasy-2022-5-0124/).

### Inclusion criteria

The inclusion criteria were as follows: (i) the study population was aged 3–18 years with NTDC; (ii) at least one of the GMSs was objectively measured and reported separately; (iii) the intervention in the study was conducted using an AVG platform, all the devices were included, (like mobile phones, virtual reality, computer games, devices like Wii, Xbox…); (iv) the study was published and peer-reviewed in English or Chinese; and (v) the study was a randomized controlled trial (RCT).

### Exclusion criteria

The exclusion criteria were as follows: (i) the type of disorder was not explicitly mentioned; (ii) evaluation of motor skill was a combination of gross motor skill and fine motor skill; and (iii) pre and post-test data on the change in GMS (e.g., mean ± SD) were absent.

### Outcome indicators

Outcome indicators selected various scales and testing indicators for evaluating GMS and three components, including the Movement Assessment Battery for Children-2 (MABC-2), Berg Balance Scale (BBS), Pediatric Balance Scale (PBS), Timed Get Up and Go Test (TUGT), Center of Pressure (COP), Functional Forward Reach Test (FFRT); Bruininks-Oseretsky Test of Motor Proficiency (BOT), Quality of Upper Extremity Skill Test (QUEST), 10 Step Test, Tracking Task (TT) and 20 Meter Shuttle Run Test.

### Literature retrieval strategy

The following databases were searched: PubMed, Cochrane Library, Embase, Elton Bryson Stephens Company, Web of Science, China National Knowledge Infrastructure, and Wanfang. We retrieved data from RCTs from the inception of each database until March 16, 2021.

The search strategy was based on principles of PICOS (population, intervention, comparison, outcomes, and study design) [24]. The search terms and expressions are as follows (taking PubMed as an example):

#1 TS = (“active video gam*” OR “active videogam*” OR “exergam*” OR “virtual realit*” OR “virtual therap*” OR “virtual environment*” OR “video gam*” OR “computer gam*” OR “serious gam*” OR “Wii” OR “Kinect” OR “PlayStation” OR “EyeToy” OR “GestureTek” OR “IREX”).

#2 TS = (“gross motor” OR “motor coordination” OR “motor skill” OR “movement skill” OR “fundamental motor skill” OR “motion capture” OR “locomotor skill*” OR “object control skill*” OR “ball skill” OR “non-locomotor skill*” OR “balance skill*” OR “stability skill*”).

#3 TS = (“Cerebral Palsy” OR “CP” OR “motor skills disorder” OR “developmental delay” OR “DCD” OR “Developmental Coordination Disorder” OR “Coordination Disorder” OR “Down Syndrome” OR “DS” OR “Autism Spectrum Disorder” OR “ASD”).

#4 TS = (“Child*” OR “boys and girls*” OR “student” OR “youth” OR “teen” OR “young person” OR “preschool” OR “adolescent”).

#5 #1 AND #2 AND #3 AND #4

### Literature screening

Two researchers used independent double-blind methods to screen the literature based on the inclusion and exclusion criteria stated above, and relevant data were extracted. If there was a disagreement in the review, screening, and data-extraction stages, a third researcher was consulted [25].

### Data extraction

The data extracted from the literature were the author names, year of publication, and basic characteristics of the samples (gaming platform, game type, outcome indicators, and intervention environment/period/duration/frequency) (Table 1).

**Table 1:** List of basic characteristics of the included documents

Researchers	Subjects		Disease Types	Intervention Setting	Supervision	AVG Platform	AVG Category	Control group	Intervention			Outcome Indicators	GMS	Risk of Bias
	E/C	Age (y)							Cycle /week	Single Time /min	Frequency /per week			
Alsaif [26]	20/20	6–10	CP	Home	Unreported	Nintendo Wii Fit	Unreported	Non-intervention	12	20	7	MABC, BOT-2		
Arnoni [27]	7/8	5–14	CP	Physical Therapy Department	Therapist	Xbox 360 Kinect	Jumping,Loading exercises	Regular Exercise	8	45	2	GMFM-88, BSA		
Bonney [28]	21/22	13–16	DCD	School	Therapist	Nintendo Wii Console and balance board	Aerobics, Muscle workout, Balance,Yoga	Task-oriented Functional Training	14	45	1	MABC, 10*5 MST		
Chen [29]	15/15	3–6	CP	Medical Clinic	Unreported	Q4 Scene Interactive Training System	Billiard Ball, Hopscotch	Regular Exercise	12	30	5	BBS, GMFM-88		
Chen [30]	20/20	3–6	CP	Medical Clinic	Unreported	Q4 Scene Interactive Training System	Billiard Ball, Hopscotch	Regular Exercise	12	40	5	BBS, GMFM-88		
Chiu [31]	30/27	6–13	CP	Home	Therapist + Parents	Nintendo Wii Sports	Bowling, Aerial sports, Frisbee and Basketball	Regular treatment	6	40	3	TT		
Cho [32]	9/9	4–16	CP	Medical Clinic	Therapist	Nintendo Wii Fit Plus	Virtual Reality Treadmill Training	Treadmill Training	8	30	3	GMFM, PBS		
Mombarg [33]	15/14	7–12	BP	School	Unreported	Nintendo Wii Balance Board	Ski-jump, Segway circuit, Obstacle course,Skate boarding	Non-intervention	6	30	3	MABC2, BOT-2		
Neto [34]	16/16	7–10	DCD	Medical Clinic	Therapist	Nintendo Wii Console and Balance Board	Table Tennis, Frisbee, Archery, Bowling,Tightrope walk and Marble balance	Task-Specific matched Training	8	60	2	MABC2		
Pourazar [35]	10/10	7–12	CP	Medical Clinic	Unreported	Xbox 360 Kinect	Dance rehabilitation training	Regular treatment	6	85–100	1	SEBT		
Ren [36]	19/16	3–6	CP	Medical Clinic	Therapist	Q4 Scene Interactive Training System	Unreported	Conventional training	12	40	5	BBS, GMFM-88		
Rojas [37]	16/16	7–14	CP	Rehabilitation centre	Unreported	Nintendo Wii Balance Board	Snowboard, Penguin Slide, Super Hula Hoop,Yoga	Standard Physiotherapy	6	30	3	COP		
Sahin [38]	30/30	7–16	CP	Pediatric clinic	Unreported	Xbox 360 Kinect	Air challenge, Boxing trainer, Wall breaker, Jet run,Super kick	Traditional occupational therapy	8	45	2	BOTMP		
Salem [21]	20/20	3–5	DCD	Medical Clinic	Therapist	Nintendo Wii Sports and Fit	Balance, Strength, Aerobics	Routine Physiotherapy	10	30	2	10WT, TUGT, GMFM		
Tarakci [39]	15/15	5–18	CP	Rehabilitation Centre	Unreported	Nintendo Wii balance	Balance-based video games	Conventional Balance Training	12	50	2	FFRT, 10WT		
Urgen [40]	15/15	7–14	CP	Unreported	Unreported	Nintendo Wii Fit	balance, weight transfer, coordination	Routine Physiotherapy and Rehabilitation	9	45	2	GMFM, PBS, TUGT		
Uysal [41]	12/12	6–14	CP	Rehabilitation centre	Therapist	Nintendo Wii Balance	Basketball, Tennis, Boxing	Routine Physiotherapy	12	30	2	PBS		
Zhang [42]	20/20	3–6	CP	Rehabilitation centre	Unreported	KMC1	Cycling game	Regular treatment	12	20	5	GMFM-88		
Zhao [43]	21/21	3–6	CP	Rehabilitation centre	Unreported	Xbox 360 Kinect	Boxing, Javelin bowling, Universe bubble ball,Bounce ball	Regular treatment	3	40	5	GMFM-88, QUEST		
Zhao [44]	21/21	3–6	CP	Rehabilitation centre	Therapist	Xbox 360 Kinect	Dance music imitation	Regular treatment	3	40	5	GMFM-88, PBS		

E = experimental group; C = control group; CP = cerebral palsy; DCD = developmental coordination disorder; MABC-2 = Movement Assessment Battery for Children-2; BBS = Berg Balance Scale; TT = Tracking Task; PBS = Pediatric Balance Scale; TUGT = Timed Get Up and Go Test; COP = Center of Pressure; FFRT = Functional Forward Reach Test; BOT = Bruininks–Oseretsky Test of Motor Proficiency; QUEST = Quality of Upper Extremity Skill Test; 10ST = 10 Step Test; 20mSRT = 20 Meter Shuttle Run Test;10*5MST = 10 *5 m sprint test-straight ①Locomotor Skills; ②Object Control Skills; ③Non-locomotor Skills

:Low risk;  :Some concerns;  : High risk

### Risk of bias in individual studies

Two researchers independently judged the risk degree of the literature according to the revised Cochrane risk of bias tool for randomised trials (RoB 2) [45]. RoB 2 sets up five bias domains: randomization process, deviations from intended interventions, missing outcome data, measurement of the outcome, selection of the reported result. There are multiple different signal problems under each domain, and each signal problem generally have five answers: Yes (Y), Probably Yes (PY), Probably No (PN), No (N), No Information (NI). The risk of bias in each domains was classified into three grades, based on participants’ assessment to signaling questions: “low risk of bias,” “some concerns” and “high risk of bias.” If all domains were rated as low risk, the study was considered as “low risk of bias”. If the assessment included one or more domains of some concerns but no high risk, the study was classified as having “some concerns”. A study was categorized as “high risk of bias”, if one or more domains were found to be at high risk. In addition, RoB 2 gave predicted direction of bias for each domains to judge the size and direction of bias: Favours experimental, Favours comparator, Towards null, Away from null, Unpredictable, NA.

### Statistical analyses

We employed Review Manager 5.4 for data processing. The cut off values for “small”, “medium”, and “large” effect sizes were 0.2, 0.5, and 0.8, respectively [46]. Additionally, 75%, 50%, and 25% denoted the proportion of “high”, “medium” and “low” interstudy heterogeneity, respectively [47]. If significant heterogeneity between studies was not observed (*P* > 0.1, *I*^*2*^ < 40%), we used a fixed effects model for analysis. If there was significant heterogeneity between studies (*P* < 0.1, *I*^*2*^ ≥ 40%), a random effects model was used for analyses, and further subgroup analyses were carried out to identify the source of heterogeneity.

If ≥ 2 tasks were used to measure the GMS of NTDC, the effect size was selected from the most commonly used tasks [48]; if the study reported multiple measurements on the same task (e.g., the ability to balance in the left, right, front, and back directions), the standard deviation and variance were averaged to represent the outcome of the task [49].

## Results

**Fig. 1:**
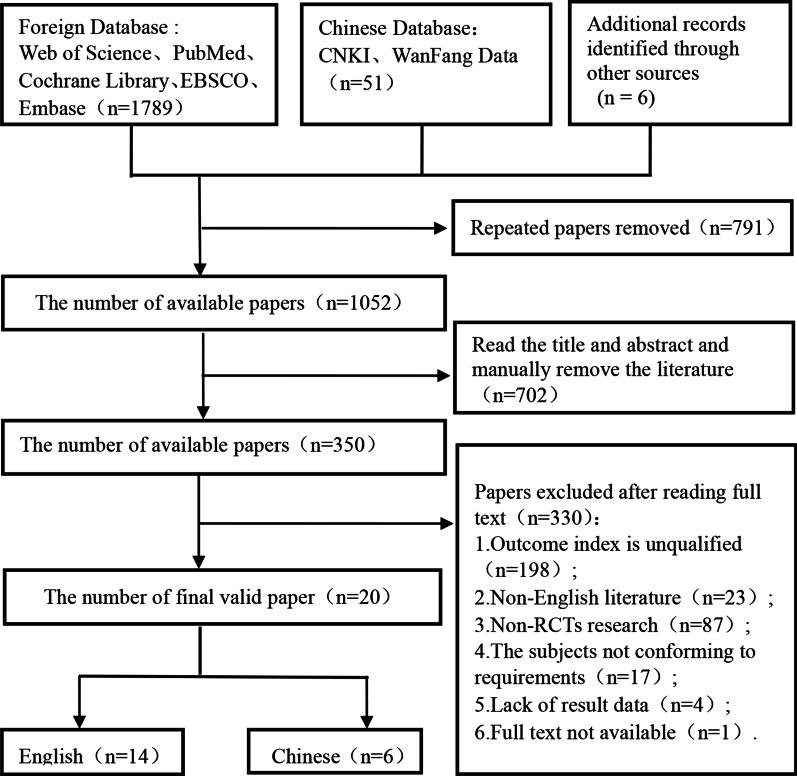
The flow chart of literature screening

A total of 1846 Chinese and English studies were obtained from seven Chinese and English databases. According to the inclusion and exclusion criteria stated above, 20 studies using RCTs were included: 14 were written in English, and 6 were written in Chinese (see Fig. 1). There were 14 studies on LS [21, 27–30, 32, 33, 36, 38–40, 42–44], 5 studies on OCS [26, 28, 31, 34, 43], and 17 studies on NLS [21, 26–30, 32–41, 44]. Among them, one article [28] evaluated 3 dimensions of GMS, and 14 articles evaluated 2 dimensions [21, 26, 27, 29, 30, 32–34, 36, 38–40, 43, 44] (see Table 1).

Interventions with AVGs among NTDC aged 3–18 for mainly focused on CP (16 items) and DCD (3 items). The game platforms included Nintendo, 360Kinect and Q4, and Nintendo Wii games were most commonly used. The game contents covered almost all sports and highlighted the fun of the game. The control group usually adopted conventional treatment or no intervention, and three articles [28, 34, 37] studied the difference between AVG and other intervention methods.

Nine studies [21, 27, 28, 31, 32, 34, 36, 41, 44] mentioned that there were therapists to supervise and guide the intervention process clearly, and the results of the subgroup analysis also showed that the intervention effect of this part was more effective. The other 11 studies did not explicitly mention the supervision of the intervention process, so it was impossible to compare the effect of supervision. In terms of intervention dose, the intervention period ranged from 3 to 12 weeks, the weekly intervention frequency ranged from 1 to 7 times, and the duration of a single intervention ranged from 20 to 100 min. However, almost all the studies did not examine exercise intensity, only Cho et al. [32] mentioned moderate exercise intensity in the intervention, so there is a lack of understanding in this regard.

### Risk of bias analysis of the included literature

As shown in Table 1 and 2, eleven studies [26–31, 38, 41–44] were rated to be at low risk of bias, eight studies [21, 32–35, 37, 39, 40] were rated as having some concerns and one study [36] was rated to be at high risk. The domain “randomization process”was classified as some concerns in seven studies [21, 32, 34–37, 40] due to these studies only mentioned allocation sequence were randomized, but did not describe the specific method of randomization. In the study of Tarakci et al. [39], the domain “missing outcome data”was rated as having some concerns due to a high drop-out rate. As the drop-out rate was same in experimental group and control group, this domain was rated with some concerns. In contrast, in the study of Ren et al. [36], the domain “missing outcome data”was judged to be at high risk due to the drop-out rate was not same in both groups (experimental group 5%, control group 20%), and it was difficult to judge whether the missingness outcome affects the true value. There were four studies [21, 33, 35, 38] was classified as some concerns in the domain “selection of the reported result” due to no study protocol or registration to compare predefined analysis intentions with reported outcomes, so whether the data that produced this result analysed in accordance with a pre-specified analysis plan that was finalized was also difficult to judge.

**Table 2:** Risk of bias in included studies

Study	Randomization process	Deviations from intended interventions	Missing outcome data	Measurement of the outcome	Selection of the reported result	Overall
Alsaif [26]						
Arnoni [27]						
Bonney [28]						
Chen [29]						
Chen [30]						
Chiu [31]						
Cho [32]						
Mombarg [33]						
Neto [34]						
Pourazar [35]						
Ren [36]						
Rojas [37]						
Sahin [38]						
Salem [21]						
Tarakci [39]						
Urgen [40]						
Uysal [41]						
Zhang [42]						
Zhao(a) [43]						
Zhao(b) [44]						

: Low risk;  : Some concerns;  : High risk

### Meta-analysis of the intervention effects of AVGs on LS in NTDC

**Fig. 2:**
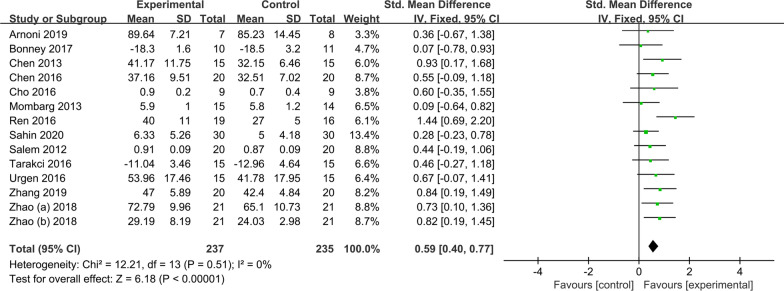
Effects of AVGs on the LS of NTDC

Fourteen RCTs were included in the meta-analysis examining the effect of AVGs on the LS of NTDC, including 472 subjects. Figure 2 shows that AVG had a significant effect on the LS of NTDC (SMD = 0.59, *P* < 0.01) compared with the control group.

A subgroup analysis (see Table 3) was conducted to further explore the differences in gaming platform, disease type, intervention setting, intervention cycle, duration of single intervention, intervention frequency, and so on. Except for the Nintendo Wii Balance Board gaming platform, children with DCD and BP, and instruction in a school setting, all subgroups showed that AVG interventions improved LS in NTDC (*P* < 0.05). In terms of the exercise dose, although there were differences between studies, the overall effect of the intervention was significant. If there is a special therapist to supervise and guide the whole process, the effect of intervention will be better.

**Table 3:** Subgroup analyses of the intervention effects of AVGs on LS of NTDC

Moderator variable	Subgroup	Included literature	*I*^*2*^	Effect size	95% CI	Two-tailed test	
						Z	*P*
Gaming platform	Nintendo Wii Sports and Fit	3	0%	0.55	(0.12, 0.97)	2.51	0.01^*^
	Nintendo Wii Balance Board	3	0%	0.22	(− 0.22, 0.66)	0.98	0.33
	Xbox™ 360	4	0%	0.54	(0.22, 0.86)	3.32	< 0.01^**^
	Q4 Scene Interactive Training System	3	37%	0.92	(0.51, 1.33)	4.41	< 0.01^**^
	KMC1	1	–	0.84	(0.19, 1.49)	2.53	0.01^*^
Control group intervention	Regular Exercise	13	0%	0.61	(0.42, 0.80)	6.29	< 0.01^**^
	Others	1	–	0.59	(0.40, 0.77)	0.17	0.86
Disease type	CP	11	0%	0.67	(0.47, 0.88)	6.35	< 0.01^**^
	DCD	2	0%	0.31	(− 0.20, 0.82)	1.20	0.23
	BP	1	–	0.09	(− 0.64, 0.82)	0.24	0.81
Intervention setting	School	2	0%	0.08	(− 0.47, 0.64)	0.29	0.77
	Medical institutions	10	0%	0.66	(0.45, 0.87)	6.20	< 0.01^*^
Intervention cycle	≤ 8 weeks	6	0%	0.48	(0.20, 0.76)	3.36	< 0.01^*^
	9–12 weeks	8	8%	0.67	(0.41, 0.94)	5.07	< 0.01^**^
Duration of single intervention	≤ 30 min	5	0%	0.57	(0.25, 0.90)	3.49	< 0.01^**^
	≥ 35 min	9	0%	0.47	(0.24, 0.69)	4.03	< 0.01^**^
Intervention frequency	< 3 times/week	6	0%	0.38	(0.09, 0.66)	2.61	< 0.01^**^
	3-5times/week	8	0%	0.75	(0.50, 0.99)	5.92	< 0.01^**^
Supervision	Yes	6	29%	0.67	(0.35, 0.98)	4.17	< 0.01^**^
	Unreported	8	0%	0.54	(0.31, 0.78)	4.60	< 0.01^**^

Bold indicate the significant values (*:p < 0.05; **:p < 0.01)

### Meta-analysis of the intervention effects of AVGs on OCS in NTDC

**Fig. 3:**
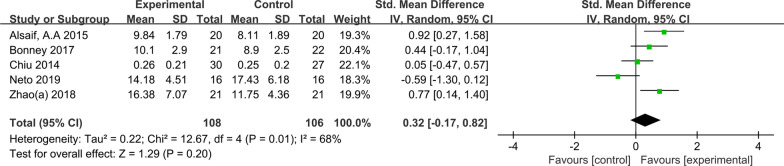
Effects of AVGs on the OCS of NTDC

Five studies reported the intervention effect of AVGs on the OCS of NTDC (see Fig. 3). The meta-analysis showed that the difference was not statistically significant (SMD = 0.32, *P* = 0.20). This indicates that AVGs did not significantly improve the OCS of NTDC compared with the control group.

### Meta-analysis of the intervention effects of AVGs on NLSs in NTDC

**Fig. 4:**
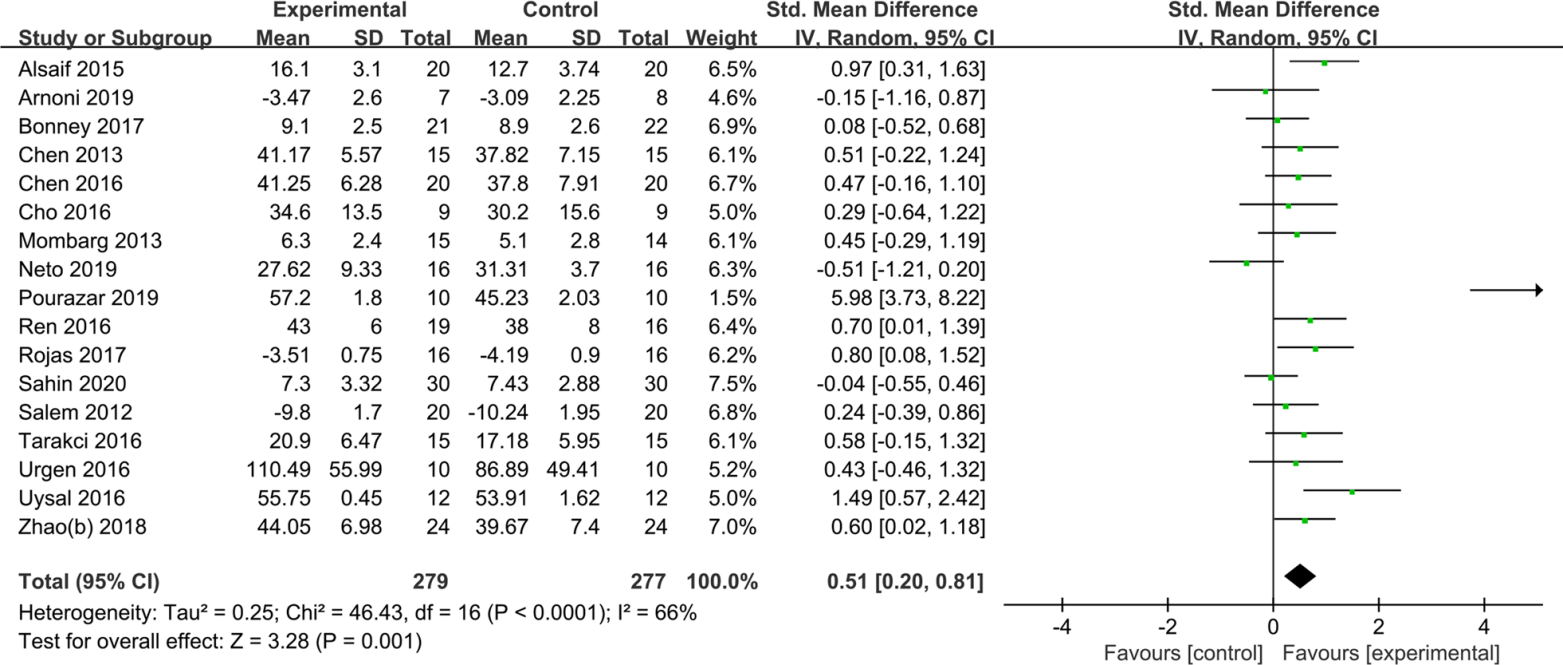
Effects of AVGs on the NLS of NTDC

Seventeen randomized controlled experiments were included in the study on the intervention of AVGs on NLS of NTDC, including 556 subjects (see Fig. 4), and the results indicated that AVGs could significantly improve NLS of NTDC compared with the control group (SMD = 0.51, *P* < 0.01).

The heterogeneity was medium (*I*^*2*^ = 66%), and the subgroup analysis was performed on gaming platform, disease type, intervention setting, intervention cycle, duration of single intervention, intervention frequency and so on to further explore the sources of potential heterogeneity (see Table 4). Table 3 shows that the Nintendo Wii and Q4 Scene Interactive Training System was conducted in a medical institution, the intervention cycle was 9–12 weeks, the duration of a single intervention was ≤ 30 min, and supervision by a therapist or parent had a significant effect on improving the NLS of NTDC. There was little difference in the intervention frequency per week.

**Table 4:** Subgroup analyses of the intervention effects of AVGs on NLS of NTDC

Moderator variable	Subgroup	Included literature	*I*^*2*^	Effect size	95% CI	Two-tailed test	
						Z	*P*
Gaming platform	Nintendo Wii Sports and Fit	4	0%	0.51	(0.14, 0.88)	2.69	< 0.01^**^
	Nintendo Wii Balance Board	6	65%	0.38	(0.08, 0.67)	2.52	0.01^*^
	Xbox™ 360	4	89%	1.05	(− 0.20, 2.31)	1.65	0.10
	Q4 Scene Interactive Training System	3	0%	0.56	(0.17, 0.95)	2.79	< 0.01^**^
Control group intervention	Regular Exercise	15	62%	0.61	(0.30, 0.93)	3.80	< 0.01^**^
	Others	2	35%	− 0.17	(− 0.62, 0.29)	0.72	0.47
Disease type	CP	13	68%	0.71	(0.33, 1.08)	3.71	< 0.01^**^
	DCD	3	0%	− 0.10	(− 0.47, 0.27)	0.52	0.60
	BP	1	–	0.45	(− 0.29, 1.19)	1.19	0.23
Intervention setting	School and Home	3	48%	0.49	(− 0.05, 1.02)	1.79	0.07
	Medical institutions	13	74%	0.56	(0.16, 0.97)	2.72	< 0.01^**^
Intervention cycle	≤ 8 weeks	8	80%	0.51	(− 0.10, 1.13)	1.63	0.10
	9–12 weeks	9	35%	0.55	(0.32, 0.79)	4.64	< 0.01^**^
Duration of single intervention	≤ 30 min	7	33%	0.60	(0.32, 0.88)	4.21	< 0.01^**^
	≥ 40 min	10	75%	0.43	(− 0.03, 0.89)	1.84	0.07
Intervention frequency	≤ 3 times/week	12	75%	0.52	(0.05, 0.99)	2.17	0.03^*^
	> 3 times/week	5	0%	0.65	(0.36, 0.94)	4.36	< 0.01^**^
Supervision	Unreported	9	73%	0.33	(− 0.05, 0.71)	1.70	0.09
	Yes	8	54%	0.71	(0.23, 1.19)	2.89	< 0.01^**^

Bold indicate the significant values (*:p < 0.05; **:p < 0.01)

## Discussion

This review aimed to examine the intervention effects of AVGs on GMS development in NTDC. Twenty RCTs were included in our meta-analysis, of which 14 were related to LS, 5 to OCS, and 17 to NLS. Overall, AVGs showed the effectiveness of intervention on GMS in NTDC and are considered a promising intervention approach for skill improvement; however, for the three subordinate concepts of GMS, there were some differences in the intervention effect of AVGs.

### Analyses of the intervention effect of AVGs on LS of NTDC

The results of this study prove that AVG could significantly improve the LS of NTDC. NTDC often present with developmental delay, poor balance, and coordination of movements, and lower-limb function disorders are often more serious than upper-limb function disorders [42]. Most of them can obtain the walking function, but the lower limb muscle, especially the tension of ankle plantar flexor muscle increases when walking, the heel cannot land completely, which is an important reason that affects the function of lower limb walking, running and jumping. Therefore, reducing the muscle tension of the ankle plantar flexor and relieving muscle spasms are very important for lower limb walking, running, jumping and daily living activities of NTDC [50].

The improvement of ankle flexibility and stability by AVG is considered to be the main reason for improving the LS of NTDC. By providing immediate feedback, virtual reality environments can elicit multisensory interactions that motivate and engage patients in longer and more intensive sessions [12]. This will undoubtedly increase the input of proprioception and improve the control ability, balance ability and coordination ability of lower limb movement. In video game training, the standing posture is often used to complete many weight fluctuation control, standing squatting, standing sitting, and other exercises, which require constant weight transfer between the lower limbs. This had a significant impact on the participants' lower limbs and maintained or expanded the range of motion of the joints, reduced the spasticity of the lower limbs, and improved the motor function of the NTDC’s lower limbs. Additionally, when the control ability of ankle joint movement is enhanced, the walking function of the child, whether in the support phase or the swing phase, tends to be more stable, thus improving walking speed. The improvement of the walking speed means the reduction of energy consumption [51], which is of great benefit to the child to save physical strength.

This study also found that the improvement of LS varied by AVG device. Wii balance boards cannot effectively improve the LS of NTDC. The game types of balance boards mainly focus on strengthening balance ability, and participants almost did not move when playing the game, so the intervention effect on the LS was not effective (*P* = 0.33). However, the other four interventions using Nintendo Wii Sports and Fit as the platform mainly used game types such as treadmill training, tennis, boxing, and frisbee. These games could continuously stimulate and strengthen the LS of the participants, resulting in a better intervention effect (*P* = 0.01).

### Analyses of the intervention effect of AVGs on OCS of NTDC

There is a general lack of research on how AVG interferes with the OCS of NTDC, and recent studies have shown varying results [2]. There were only 5 studies on OCS included in this study, and the intervention effect was limited, which does not support its significant improvement in OCS.

The physical activities in these games include motor tasks that involve a wide range of sensory feedback, and visual feedback is dominant [52]. Although AVGs can simulate rich real-world scenes, tactile stimulation is difficult to fully practise and develop in this simulation environment [11]. Tactile is the feeling produced when contacting external stimuli, which is different from LS and NLS; they require tactile stimulation to provide real-world experience, require upper or lower limbs to contact objects for object control, and perform actions such as throwing, slapping, and kicking. In this process, the touch between the body and object plays an important role, which is difficult to replicate in virtual reality technology. Neither the game handle in hand nor the controller worn on the body can provide timely haptic feedback, such as the weight and size of the control object. Therefore, some scholars began to propose using haptic feedback gloves when using video games to simulate ball operations in real life. By wearing gloves, participants can feed back more haptic information in a timely manner to improve the intervention effect of AVGs on OCS [53].

Although the overall effect of AVGs on improving OCS in this study was not significant, Chiu et al. [31] showed that the range and frequency of use of children's upper limbs have a significant increase compared with the past after video game intervention, which greatly improves their independence level in daily activities [38]. This undoubtedly has an important impact on the development and improvement of upper limb function in NTDC.

### Analyses of the intervention effect of AVGs on NLSs of NTDC

The Nintendo Wii platform was the most utilized among the interventions of NLS, Balance Board and Wii Sports and Fit and was equally effective in the balance intervention. The AVG platforms could more sufficiently replicate real-world balance skills compared with other types of GMS due to the designed method [53], and this may be the reason why AVG interventions have an effect on balance.

Visual feedback theory provides theoretical hypotheses for video games boosting participants' balance skills. The theory holds that when playing video games, children can see their actions on the video screen immediately, which constitutes a new effective learning method, implicit learning [9]. The tasks practised during video games incorporate a wide range of visual–perceptual processing [52]. The visual timely feedback enables the participants to continuously adjust and control the position of the body during the game. Once the child initially learned to maintain equilibrium on items, more challenging dynamic tasks, such as jumping, striking and catching balls, were introduced. It enhanced the frequency and intensity of visual feedback, allowing participants to continuously perform posture detection and balance disturbance correction in response to different balance conditions. Additionally, the game exercises completed in the standing position increased the stability of the participants' trunk, the symmetry on both sides of the body was improved, the centre of gravity of the body was evenly distributed on the lower limbs, the stability of standing was increased, and the ability of posture control was improved [32, 54].

It is worth noting that when using AVGs to intervene in the NLS of NTDC, attention should be given to the control of exercise intensity and trying to avoid heavy exercise in a short time or a long-term balance exercises. Ruzic et al. [55] found that high-intensity exhaustive exercise load has a negative impact on both static and dynamic balance ability after studying the relationship between exercise load and balance ability with healthy people as samples. Although no similar study has been conducted on NTDC, it deserves our attention.

### Moderating variable analysis of the intervention effect of AVGs on the GMS of NTDC

In this study, there was no significant difference in the improvement of OCS; therefore, only the LS and NLS subgroups were analysed.

The subgroup analysis of the intervention effect shows that the effect of AVG intervention was likely to be related to the type of game being intervened. The game platform of Nintendo Wii Sports and Fit, Xbox™ 360 and Q4 in LS, Wii Sports and Fit, Q4 and Nintendo Wii Balance Board in NLS all produced significant intervention effects. In the LS intervention, there were four studies using Wii balance boards. Game types mainly focus on strengthening balance ability, including skate boarding, skiing game, Yoga, and ski-jump, and the intervention effect of such games is not significant on LS. Studies also showed that the Q4 platform has a significant intervention effect in LS and NLS, but considering that the studies of the Q4 game platform mainly come from Chinese scholars, the results were relatively limited, and the intervention with other countries and ethnic groups needs further demonstration.

Similarly, the mismatch between game types and motor skills was likely to be the main reason for the ineffectiveness of interventions in the DCD population and in school settings. Balance boards were used in two of the three DCD population studies (*P* = 0.23) and were used in all three school setting studies (*P* = 0.77). Therefore, appropriate game equipment and contents should be selected according to specific needs in future intervention practices.

In the past, few studies have discussed the ideal frequency of AVG intervention in motor skill development because other characteristics included in the study are different, which will also lead to some differences in intervention results. Therefore, it is difficult to identify the specific and scientific intervention doses. In terms of the exercise dose of the intervention, there was little difference among different intervention periods, intervention frequencies and single intervention times. The conclusion is consistent in improving LS and NLS. The ideal AVG-based intervention protocol was found to be > 3 times a week, duration of single intervention ≤ 30 min, and a total duration of 9–12 weeks.

As another important aspect of exercise dose, exercise intensity was rarely mentioned in the study of AVG intervention whether in LS or NLS with NTDC. Only one of the 20 included studies mentioned that the intervention intensity was moderate [32]. A safe, enjoyable, and playful environment is associated with less fatigue and more relaxation [19]. Therefore, the actual exercise intensity of AVG may be greater than that of traditional exercise therapy. However, it was difficult to draw clear conclusions as to which exercise intensity was the most appropriate for developing the LS or NIS of NTDC.

Professional supervision and guidance during the intervention process would also have an important impact on the effect. When children play sports video games without any guidance, their skill execution ability is poor [56]. In this study, 6 studies in LS intervention and 8 studies in NLS explicitly mentioned that the intervention process was supervised by a therapist, and the intervention was effective.

The effects of CP and intervention in medical institutions were more effective than DCD and intervention at home and school. The latter was examined in a small number of relevant studies, so the effects are uncertain. Compared with traditional balanced rehabilitation treatment, the intervention effect of AVG is stronger, but AVG cannot be considered the most effective method of LS or NLS intervention. Neuromotor task training [57] and task-specific matched training [34] in NTDC were proven to be more effective than AVG intervention. The choice of AVG may be a comprehensive decision considering the cost of use, the convenience of the equipment, the fun of the game and the popularity of promotion.

The strength of this study was that it comprehensively analysed the intervention effect of AVGs on GMS with NTDC and discussed the three components of GMS, but the study is not without limitations. And the outcomes in the systematic review are limited by the following aspects. To begin, although the included research subjects are all non-typically developing children and adolescents that have certain motor skills development disorders, the pathogenesis of different disease types is not the same, and the degrees of disease of the same disease type were not subdivided, which causes there are vast differences in the original motor capabilities and required intervention of the intervention subjects [2], so the intervention effect of AVGs may reach different conclusions. Second, most of the research subjects included in this study are CP, and there are few studies on other disease types. So the intervention effect of AVGs on GMS in these children (e.g. DCD, BP) is unclear. The conclusions drawn from the studies would be more reliable and representative among non-typically developing children and adolescents if there were a sufficient number of studies involving each disease type. Third, a subgroup analysis of LS and NLS produced a relatively ideal AVG-based intervention exercise dose, but this dose did not include exercise intensity. Exercise intensity is an important component of exercise intervention, and different intensities may produce different intervention effects. Without considering exercise intensity, discussing the intervention frequency, single time of intervention and intervention cycle, the conclusions obtained will be questioned to some extent. Exercise intensity will be an important aspect that needs to be paid attention to in the future research on GMS development of NTDC with AVG intervention. Finally, despite detailed searches of relevant seven Chinese and English databases, the current review is limited by the inclusion of only Chinese and English language publications of peer-reviewed full-text. Other unpublished papers, ongoing studies, and non-English, non-Chinese work on this topic were not included, some potentially important studies may still have missed.

## Conclusions

AVGs provide a safe and interesting environment, produce less fatigue, and greater load intensity and total amount by the body, which increases the physical activity level of game participants and improves the practice effect. The results of this study show that AVG is an effective rehabilitation treatment tool for GMS intervention in NTDC. Especially in LS and NLS, the research conclusions are relatively consistent, and the intervention effects reach a medium effect. However, it is necessary to select the game type and content that match the motor skills in AVG intervention. Because the number of relevant studies is small, and the influence of OCS remains unclear.

According to the present review, the ideal protocol for a motor skills intervention is > 3 times a week, duration of single intervention ≤ 30 min, and total duration 9–12 weeks. The interventions conducted in medical settings and supervised and guided by professional therapists were most effective. However, the current research on AVG interventions among NTDC to enhance motor skill development rarely mentions exercise intensity. Intensity is an important component of exercise dose, and different exercise intensities will lead to different intervention results. This may be an important aspect that leads to differences between studies. Therefore, research on exercise intensity will be the focus of future AVG interventions for the development of motor skills.

## Data Availability

All data analysed for this review are included in this published article.

## Abbreviations

NTDC: Non-typically developing children
AVGs: Active video games
GMS: Gross motor skill
CP: Cerebral palsy
RCT: Randomized controlled trials
LS: Locomotor skills
OCS: Object control skills
NLS: Non-locomotor skills
